# Habitat suitability for the soybean aphid, *Aphis glycines*, and its natural enemies: implications for biological control and soybean protection

**DOI:** 10.3389/fpls.2026.1845163

**Published:** 2026-06-03

**Authors:** Zhengbing Wang, Mingsheng Yang, Zhen Li, Xin Miao, Jin Liu, Jiahui Zhang, Liuyong Xie, Kedong Xu, Weili Ding, Wu Zhang, Peng Dai, Keshi Ma

**Affiliations:** 1 College of Life Science and Agronomy, and Field Observation and Research Station of Green Agriculture in Dancheng County, Zhoukou Normal University, Zhoukou, Henan, China; 2 Key Laboratory of Plant Genetics and Molecular Breeding, and Henan Key Laboratory of Crop Molecular Breeding and Bioreactor, Zhoukou Normal University, Zhoukou, Henan, China; 3 Finance Office, Zhoukou Normal University, Zhoukou, Henan, China; 4 Heilongjiang Academy of Agricultural Sciences, Heihe, Heilongjiang, China; 5 Institute of Biological Control Laboratory, Jilin Agricultural University, Changchun, Jilin, China

**Keywords:** biocontrol, MaxEnt, pest management, potential suitable habitats, species distribution model

## Abstract

The soybean aphid, *Aphis glycines* Matsumura, is one of the most destructive pests affecting soybean production, frequently inflicting substantial economic losses. In this study, an optimized MaxEnt model was employed to predict the habitat suitability of *A. glycines*, its host plant *Glycine max*, and four key natural enemies (*Harmonia axyridis*, *Chrysopa pallens*, *Episyrphus balteatus*, and *Lysiphlebia japonica*). Furthermore, the overlaying ranges of suitable habitats of *A. glycines* and these natural enemies were identified. Results indicated that the area under the receiver operating characteristic (AUC) values of the six models ranged from 0.856 to 0.981 and the true skill statistic (TSS) values from 0.470 to 0.733. The highest contributing variables for the models of *A. glycines*, *G. max*, *H. axyridis*, *C. pallens*, *E. balteatus*, and *L. japonica* were BIO04 (temperature seasonality), BIO18 (precipitation of warmest quarter), BIO14 (precipitation of driest month), BIO01 (annual mean temperature), BIO18, and BIO17 (precipitation of driest quarter), respectively. Different natural enemies exhibited distinct niche overlaps with *A. glycines* in spatial distribution. For instance, the overlaying range between *A. glycines* and *H. axyridis* were distributed across East China, Korea, and Japan, whereas those between *A. glycines* and *L. japonica* were mainly concentrated in parts of South China and most of Japan. The differences suggest that priority regions for the release of different enemies can be identified to potentially control *A. glycines*. Compared with previous modeling study on *A. glycines*, this study adopted a MaxEnt parameter optimization approach and defined the suitable habitats of *A. glycines* under the presence of its host *G. max*. The results could enhance the understanding of the potential distribution of *A. glycines* and effectively facilitate the selection and application of natural enemies for regional biological control of this pest.

## Introduction

The soybean, *Glycine max* (L.) Merr., was first domesticated in China approximately 5,000 years ago and is now cultivated globally (Hymowitz, 1970; Wu et al., 2004). This crop serves as a significant source of protein for both humans and animals, as well as an essential industrial raw material (Yano et al., 2025). In soybean cultivation, pests are a critically limiting factor that often leads to yield loss and a decline in soybean quality. The impact of these pests has tended to worsen with global climate change and alterations in cropping systems (Bortolotto et al., 2015; Gao et al., 2018). Chemical control is currently a primary method used against soybean pests. Meanwhile, biological control using natural enemies is also being developed and applied due to its environmental safety and sustainable management potential (Gao et al., 2018; Wu et al., 2004).

The soybean aphid, commonly known as *Aphis glycines* Matsumura (Hemiptera: Aphididae), is one of the most destructive pests of soybean (Gao et al., 2018; Wu et al., 2004). This pest frequently causes significant yield loss in soybean crops (up to 50% during outbreak years) by extracting sap from leaves, stems, and pods, and by transmitting plant pathogens such as the soybean mosaic virus (Hill et al., 2001; Ragsdale et al., 2011, 2007; Sun et al., 2015a). *Aphis glycines* is native to East Asia, mainly across China, Korea, and Japan (Fang et al., 2018; Wu et al., 2004). In USA, it was first detected on soybean in Wisconsin in 2000, and has since rapidly adapted to the north-central states of the USA, becoming a serious pest globally (Ragsdale et al., 2011, 2004; Tilmon et al., 2011). This pest is a typical heteroecious holocyclic species, exhibiting host alternation with sexual reproduction during part of its life cycle. It uses certain buckthorn trees (e.g., *Rhamnus davurica* and *R. japonica* in China and Japan) as the primary host for sexual reproduction, and soybeans (including cultivated *Glycine max*, and wild *G. soja*) as the secondary host during asexual reproduction (Qiu et al., 2025; Ragsdale et al., 2011; Rutledge et al., 2004; Takahashi et al., 1993; Wu et al., 2004). In management practices, broad-spectrum insecticides such as pyrethroids, neonicotinoids, and organophosphates have been applied to form the basis of Integrated Pest Management (IPM) programs against this widely distributed invasive pest (Hodgson et al., 2012; Kaleem Ullah et al., 2023; Koch et al., 2016; Ragsdale et al., 2011). However, the frequent use of pesticides has led to the development of resistance in the pest and potentially severe environmental side effects, posing a serious threat to its sustainable control and food security (Koch et al., 2020; Wu et al., 2004). Therefore, environmentally safe methods, such as the use of natural enemies, have been recommended against this pest.

Investigations and evaluations of potential biological control agents against *A. glycines* have been conducted for decades through field surveys and experimental studies. It has been reported that *A. glycines* is targeted by a diverse array of natural insect enemies, primarily including predators and parasitoids (Ragsdale et al., 2011; Rutledge et al., 2004). Predators are commonly found in soybean fields in East Asia include ladybugs, lacewings, and predatory flies, represented by *Harmonia axyridis* (Pallas), *Chrysopa pallens* (Rambur), and *Episyrphus balteatus* (de Geer), respectively (Dai and Zu, 1997; Li et al., 2000; Liu and Zhao, 2007; Van Den Berg et al., 1997; Wu et al., 2004). Among parasitoids, *Lysiphlebia japonica* (Ashmead) is often recorded as an effective enemy against *A. glycines* (Liu and Zhao, 2007). In laboratory or field experiments, *H. axyridis*, in particular, has proven highly effective in managing *A. glycines* (Liu et al., 2012; Sun et al., 2015b). The biocontrol system is complex, and one of the important factors affecting biocontrol effectiveness is the selection of biological control agents within a certain range, because the habitats with environmental conditions that optimally match the ecological requirements of certain enemies, such as temperature and precipitation, are ideal for biological control (Hoelmer and Kirk, 2005; Zhao et al., 2023). Therefore, to enhance the effectiveness of natural enemies against pests, establishing the overlaying ranges of pests and enemies through the distribution predictions of suitable habitats is crucial for biological control and has garnered significant interest in the context of integrated pest management (Harman et al., 2025; Li et al., 2023; Lian et al., 2022; Mukherjee et al., 2011; Sun et al., 2017; Zhang et al., 2025; Zhao et al., 2023).

Species distribution model (SDM) is a very popular tool in ecology and biogeography, which link the ecological requirements of a species through known distribution points and environmental predictor layers to project the potential habitat distribution of this species (Elith and Leathwick, 2009; Yang et al., 2024b; Zhu et al., 2013). In SDM practice, there are various modeling methods, such as the maximum entropy (Maxent) method that uses a machine-learning algorithm based on the principle of maximum entropy to predict the habitat suitability of an indicated species (Phillips et al., 2017, 2006). Recently, an ensemble modeling method, which integrates a suite of different SDMs to estimate habitat suitability by consensus, has been increasingly used (Zhao et al., 2023). Alternatively, the MaxEnt method with optimized parameters has also been widely employed due to its better prediction validity with small sample size and easy interpretation of prediction results (Elith et al., 2006, 2011; Phillips et al., 2006; Sun et al., 2020; Zhang et al., 2024).

To improve biocontrol effectiveness, selecting appropriate natural enemy species determined through overlaying analyses based on SDM results has been widely conducted for many pests, as mentioned above. However, no relevant studies have been carried out on *A. glycines*. In this study, we developed SDMs for *A. glycines*, its host plant *G. max*, and four common natural enemies (*H. axyridis*, *C. pallens*, *E. balteatus*, and *L. japonica*) using the optimized MaxEnt algorithm (Phillips et al., 2006; Phillips and Dudík, 2008). We firstly identified the potential suitable habitats of *A. glycines*, taking into account the presence of host plant *G. max*. Further, through overlaying analyses, we defined the overlaying ranges of four representative natural enemies against *A. glycines*. In the modeling procedures, the key driving factors underlying the habitat distribution of each modeled species were generated. Our findings could enhance understanding of the potential distribution patterns of *A. glycines* and, more importantly, offer insights for the regional management of biological control of *A. glycines* associated with the selections of natural enemies within its native range.

## Materials and methods

### Study species

Except for *A. glycines*, its common host *G. max* was modeled to define the potential suitable habitats of *A. glycines* through overlaying analysis, considering their close ecological associations, and for the targeted management of *A. glycines* in soybean fields. In addition, from each representative group of natural enemies, such as ladybeetles, lacewings, predatory flies, and parasitoids, that have been documented as preying on *A. glycines* in prior studies (Liu and Zhao, 2007; Tilmon et al., 2011; Wu et al., 2004), one representative was selected based on the following criteria: (i) the species is common in East Asia; (ii) the species has sufficient occurrence records for modeling; (iii) the species has been reported as the natural enemies in field observations. Finally, *A. glycines*, its host *G. max*, and four natural enemies, namely *H. axyridis*, *C. pallens*, *E. balteatus*, and *L. japonica*, were used in the modeling procedures.

### Species occurrence records

**Figure 1:**
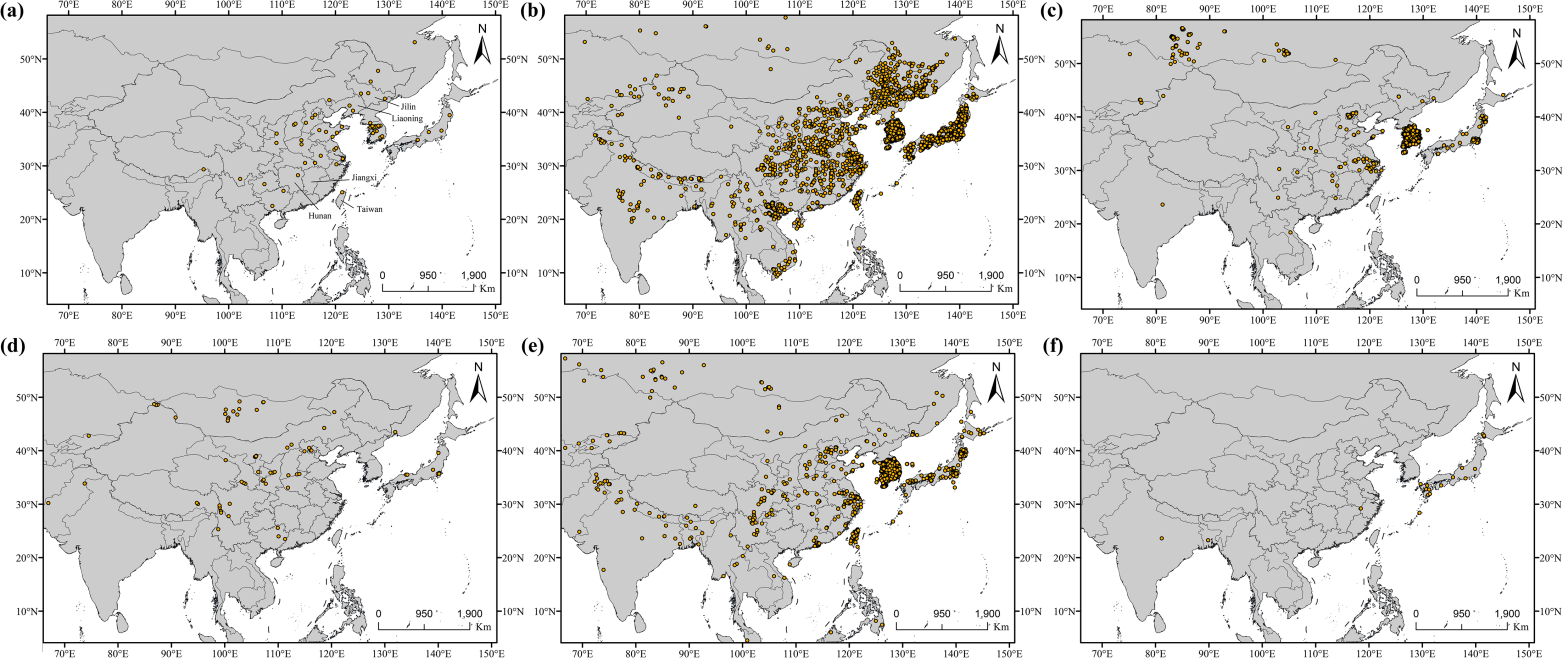
Occurrence records of the six study species used in the modeling in the study area. The dots indicate the occurrence records used in the models. **(a)** *Aphis glycines*. **(b)** *Glycine max*. **(c)** *Harmonia axyridis*. **(d)** *Chrysopa pallens*. **(e)** *Episyrphus balteatus*. **(f)** *Lysiphlebia japonica*.

The known occurrence records for each of the six species examined were primarily obtained from three sources: (i) the Global Biodiversity Information Facility (GBIF, https://www.gbif.org/species, accessed 10 August 2024); (ii) the Barcode of Life Data system (BOLD, https://www.boldsystems.org/, accessed 10 August 2024), and (iii) published literature indexed in the Web of Science (https://www.webofscience.com, accessed 22 August 2024) and the China National Knowledge Infrastructure (https://www.cnki.net, 22 August 2024). In the literature, only clear distribution localities for the target species were considered and subsequently converted into coordinate data using the website https://api.map.baidu.com/lbsapi/getpoint/. For each species, the initial occurrence records compiled from the four sources were 104 for *A. glycines*, 3835 for *G. max*, 738 for *H. axyridis*, 80 for *C. pallens*, 1565 for *E. balteatus*, and 28 for *L. japonica*. The R package ‘CoordinateCleaner’ (Zizka et al., 2019) was then utilized, employing its *clean_coordinates* function to remove ineffective occurrence records, such as those assigned to the sea, country capitals, or biodiversity institutions. Moreover, to minimize possible spatial deviation due to sampling bias (Gaul et al., 2022; Phillips et al., 2009; Zhu et al., 2014), the R package ‘spThin’ (Aiello-Lammens et al., 2015) was used to thin the coordinates, ensuring that no more than one record was present in each raster cell (approximately 4.5 km²). Finally, the occurrence records retained for modeling were 61 for *A. glycines*, 2438 for *G. max*, 668 for *H. axyridis*, 77 for *C. pallens*, 1565 for *E. balteatus*, and 22 for *L. japonica* (**Figure 1**, **Supplementary Table 1**).

### Selection of bioclimatic variables

Nineteen bioclimatic variables (**Supplementary Table 2**) representing the near-current-year periods were downloaded from WorldClim 2.1 (https://www.worldclim.org/) (Fick and Hijmans, 2017). Utilizing the coordinates (65.255 E, 56.764 N; 147.823 E, 56.764 N; 147.823 E, 5.254 N; 65.255 E, 5.254 N), the R packages ‘raster’ (Hijmans, 2023) and ‘rgeos’ (Bivand and Rundel, 2020) were employed to generate the layer of study area from the environmental layers downloaded at a global scale. Considering the potential for collinearity among variables, which could lead to model overfitting and thus diminish the prediction accuracy of the MaxEnt model (De Marco and Nóbrega, 2018; Dormann et al., 2013; Low et al., 2021), variable screening was performed. Initially, Pearson’s correlation analyses were conducted for all 19 variables using the R package ‘corrplot’ (Wei and Simko, 2021). Subsequently, an initial model for each target species was developed using Maxent software (https://biodiversityinformatics.amnh.org/open_source/maxent/) to ascertain the percentage contribution of each variable. For any two variables with a correlation coefficient |r| greater than 0.8, the one contributing less to the initial model was discarded (Phillips et al., 2006; Ran et al., 2024). In addition, variables with a percentage contribution of less than 1% were also eliminated. All environmental layers were constructed with a 2.5’ spatial resolution.

### Model setting

The MaxEnt software was utilized to develop the distribution model for each target species. We explicitly addressed the trade-off between model complexity and sample size in our MaxEnt framework. Given the variation in numbers of occurrence record across *A. glycines* and its natural enemies, the parameters of feature classes (FCs), regularization multipliers (RMs), and their combinations were optimized (Muscarella et al., 2014; Zhao et al., 2024) using the R packages ‘ENMeval’ (Kass et al., 2021) and ‘dismo’ (Lobo et al., 2008). In this process, six FCs (L, H, LQ, LQH, LQHP, and LQPHT) and eight RMs (ranging from 0.5 to 4.0 in increments of 0.5) were selected to calculate the standardized Akaike information criterion coefficient (AICc). The optimal combination of FC and RM, when the delta AICc score was at its lowest, was implemented in the MaxEnt procedure. In MaxEnt modeling, we randomly assigned 75% of the occurrence records of each target species as training data and the remaining 25% as test data. Ten replicates were performed for each analysis, with the maximum iterations set to 5,000 and background points set to 10,000. The options ‘create response curves’ and ‘do jackknife to measure variable importance’ were chosen to evaluate the variable contribution in MaxEnt projections.

### Model evaluation and analyses

The models’ performance was evaluated using two criteria: the area under the receiver operating characteristic (ROC) curve, known as the AUC (Higmans et al., 2017), and the true skill statistic (TSS) (Allouche et al., 2006). The AUC values were produced by Maxent software, ranging from 0 to 1, with an AUC value of 0.7−0.8 considered acceptable, 0.8−0.9 deemed great, and values greater than 0.9 considered significant (Hand and Anagnostopoulos, 2013; Peterson et al., 2008). Conversely, a value below 0.5 suggests that the model’s performance is no better than random. The TSS values were computed using the R package ‘ecospat’ (Di Cola et al., 2017), and the values generated range from −1 to +1, where a value close to 1 signifies perfect prediction, and values of zero or less indicate that the model’s performance is no better than random (Allouche et al., 2006; Pearce and Ferrier, 2000).

The prediction maps generated by Maxent software displayed continuous values of habitat suitability. To more clearly illustrate the distribution patterns of suitable habitats for the target species, we used the maximum training sensitivity plus specificity (MTSPS) logistic threshold value as a threshold value to define suitable and unsuitable areas, as has been done in other studies (Liu et al., 2016; Xue et al., 2022; Yang et al., 2024a). The MTSPS logistic threshold values generated for each species were 0.180 for *A. glycines*, 0.296 for G. max, 0.133 for *H. axyridis*, 0.401 for *C. pallens*, 0.231 for *E. balteatus*, and 0.343 for *L. japonica*. Specifically, areas on the prediction map were considered suitable when the habitat probability exceeded the threshold value. Habitats with suitability values greater than the threshold were further categorized into lowly and highly suitable areas, which were defined using the median value of the suitability values within the range between the MTSPS threshold and the highest suitability value (approaching 1). The impact or importance of variables to the modeling was evaluated by the index of percent contribution and jackknife values produced by MaxEnt procedures.

### Overlaying analyses

The overlaying analyses were carried out in two sequential stages using ArcGIS 10.4 (Esri, Redlands, California, USA): conversion of continuous suitability layers to binary layers, and spatial overlaying of binary layers. First, to facilitate the identification of suitable/unsuitable areas and subsequent overlaying calculations, continuous suitability layers of all study species were conversed to binary layers using the “Reclassify” tool (Spatial Analyst > Reclassify). Second, overlaying analysis was conducted between the binary layers of *A. glycines* and *G. max*, and the overlaying result was further overlapped with the binary layer of each of the four enemies. In the overlaying analysis, the “Intersect” tool in ArcGIS 10.4 (Analysis Tools > Overlay > Intersect) was used, because it can accurately retain the spatial areas that are suitable for both binary layers. Finally, the overlaying ranges were defined to establish the habitat coverage of *A. glycines* and enemy species at a spatial scale.

## Results

### Variable selection, model parameter, and performance

**Figure 2:**
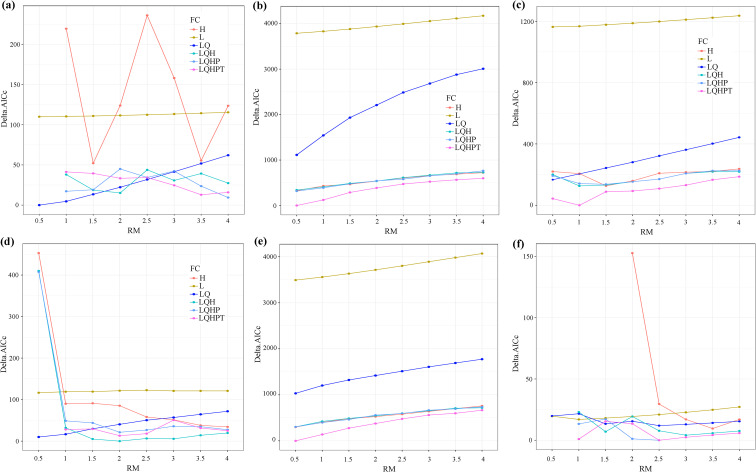
The changes of feature classes at different regularization multiplier. The parameter combinations with the lowest Delta. AICc were used in the models. FC, feature class; H, hinge; L, linear; P, product; Q, quadratic; RM, regularization multiplier; T, threshold. **(a)** *Aphis glycines*. **(b)** *Glycine max*. **(c)** *Harmonia axyridis*. **(d)** *Chrysopa pallens*. **(e)** *Episyrphus balteatus*. **(f)** *Lysiphlebia japonica*.

Analyses of Pearson’s correlation coefficient (**Supplementary Figure 1**) and initial MaxEnt models identified six of the 19 bioclimatic variables for all six study species. In detail, the retained variables were BIO02 (mean diurnal range), BIO04 (temperature seasonality), BIO08 (mean temperature of wettest quarter), BIO13 (precipitation of wettest month), BIO15 (precipitation seasonality), and BIO18 (precipitation of warmest quarter) for *A. glycines*, BIO02, BIO04, BIO10 (mean temperature of warmest quarter), BIO13, BIO14 (precipitation of driest month), and BIO18 for *G. max*, BIO03 (Isothermality), BIO08, BIO09 (mean temperature of driest quarter), BIO12 (annual precipitation), BIO14, and BIO18 for *H. axyridis*, BIO01 (annual mean temperature), BIO02, BIO03, BIO14, BIO18, and BIO19 (precipitation of coldest quarter) for *C. pallens*, BIO02, BIO08, BIO11 (mean temperature of coldest quarter), BIO14, BIO16 (precipitation of wettest quarter), and BIO18 for *E. balteatus*, and BIO02, BIO03, BIO06 (min temperature of coldest month), BIO08, BIO12, and BIO17 (precipitation of driest quarter) for *L. japonica*. The optimized RM and FC parameter combinations for the six study species were determined as follows: RM = 0.5 and FC=LQ for *A. glycines*, RM = 0.5 and FC=LQHPT for *G. max*, RM = 1 and FC=LQHPT for *H. axyridis*, RM = 2 and FC=LQH for *C. pallens*, RM = 0.5 and FC=LQHPT for *E. balteatus*, and RM = 2.5 and FC=LQHPT for *L. japonica* (**Figure 2**). The AUC and TSS values for each model are presented in **Table 1**. The AUC values ranged from 0.856 ± 0.038 to 0.981 ± 0.013, with the lowest value for *C. pallens* and the highest value for *L. japonica*. The TSS values ranged from 0.470 to 0.733, with the lowest value for *C. pallens* and the highest value for *L. japonica* as well.

**Table 1:** The area under the receiver operating characteristic curve (AUC) and the true skill statistic (TSS) values of six MaxEnt models for the six study species.

Species	AUC	TSS
*Aphis glycines*	0.919 ± 0.024	0.490
*Glycine max*	0.861 ± 0.003	0.581
*Harmonia axyridis*	0.945 ± 0.011	0.650
*Chrysopa pallens*	0.856 ± 0.038	0.470
*Episyrphus balteatus*	0.914 ± 0.005	0.718
*Lysiphlebia japonica*	0.981 ± 0.013	0.733

### Variables importance in the modeling

**Figure 3:**
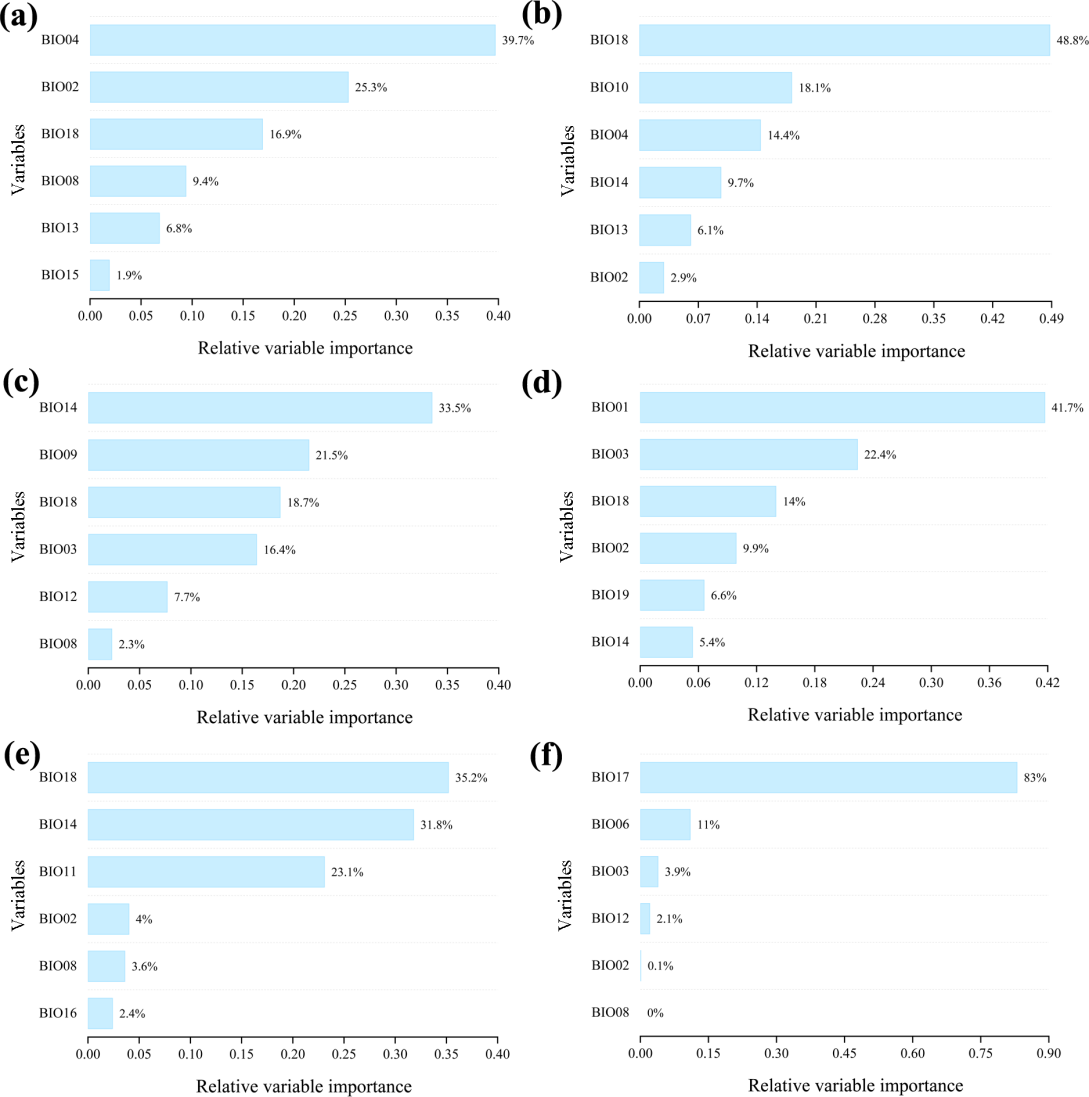
The relative percentage contributions of bioclimatic variables across six models for the six studied species. Column length indicates the magnitude of each percentage value. **(a)** *Aphis glycines*. **(b)** *Glycine max*. **(c)** *Harmonia axyridis*. **(d)** *Chrysopa pallens*. **(e)** *Episyrphus balteatus*. **(f)** *Lysiphlebia japonica*.

The contribution values, expressed as percentages of each variable in the modeling, for six study species are visualized in **Figure 3**. The variables BIO04, BIO18, BIO14, BIO01, BIO18, and BIO17 represented the highest contributing variables to the models for *A. glycines*, *G. max*, *H. axyridis*, *C. pallens*, *E. balteatus*, and *L. japonica*, respectively. In contrast, the variables BIO15, BIO02, BIO08, BIO14, BIO16, and BIO08 represented the lowest contributing variables, respectively. Notably, for *L. japonica*, BIO17 was predominant with a contribution of up to 83%, in contrast to BIO08, which had a negligible contribution. The importance of variables informed theirs close associations with the ecophysiological mechanisms of species. For instance, temperature seasonality (BIO04) affects the overwintering and reproduction of *A. glycines*, while precipitation in the warmest and driest quarters (BIO18 and BIO17) regulates the growth of the host plant *G. max* and the foraging activity of natural enemies, thereby shaping their habitat suitability patterns.

**Figure 4:**
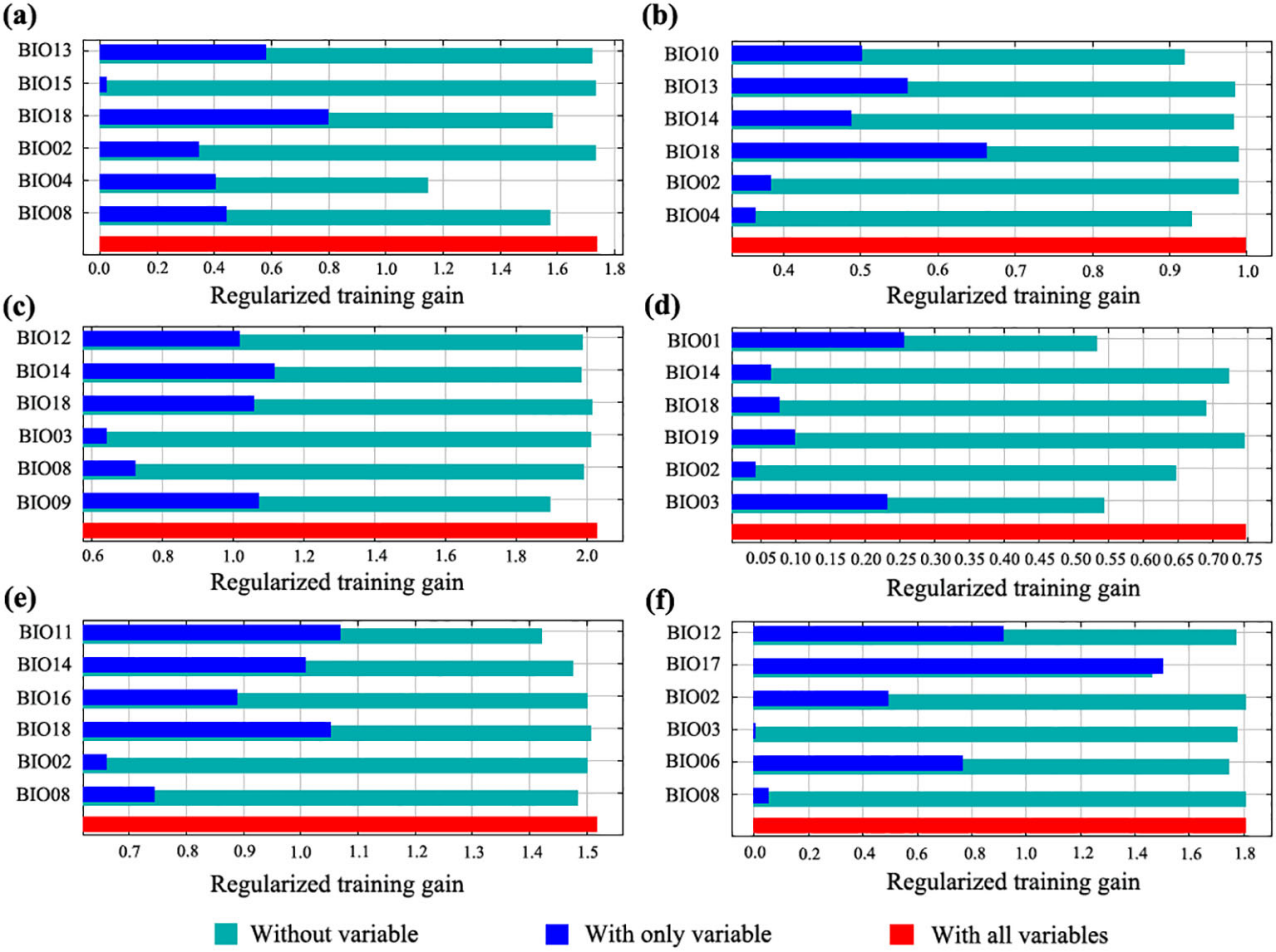
The results of jackknife tests. Variables with longer blue bar or shorter green bar are considered to have greater relative importance in the modeling. **(a)** *Aphis glycines*. **(b)** *Glycine max*. **(c)** *Harmonia axyridis*. **(d)** *Chrysopa pallens*. **(e)** *Episyrphus balteatus*. **(f)** *Lysiphlebia japonica*.

The jackknife analyses (**Figure 4**) revealed patterns of variable importance that were largely consistent with the contribution values expressed as percentages. In these analyses, a significant observation was that when considering one variable or excluding it, the variables BIO04, BIO18, BIO14, BIO01, BIO18, and BIO17 exhibited longer bar lengths, or all other variables collectively showed shorter bar lengths. This indicated that these six variables, as revealed by the contribution values, contributed more significantly, to the modeling for the respective six species.

### Potential suitable habitats of *A. glycines* and *G. max*, and their overlaying range

**Figure 5:**
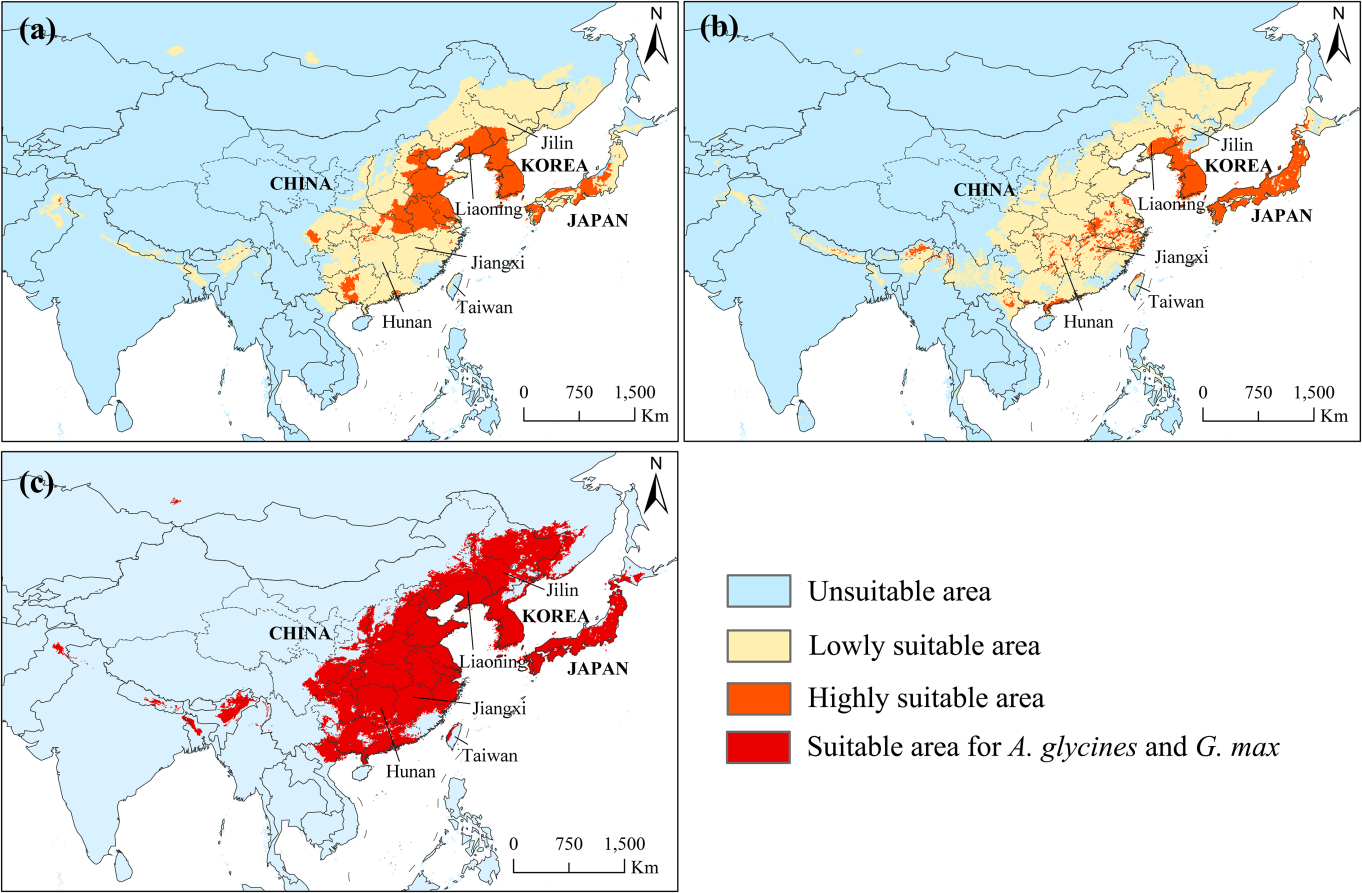
Potential suitable habitats of *Aphis glycines* and *Glycine max*, and overlaying range of their suitable habitats. Countries (marked in bold) and five Chinese provinces referenced in the text are labeled to facilitate understanding of the relevant descriptions. **(a)** *Aphis glycines*. **(b)** *Glycine max*. **(c)** *Aphis glycines* + *Glycine max*.

In its native range, the suitable habitats of *A. glycines* (with a suitability index of 0.180–1) were primarily distributed across East China, Korea, and Japan, at a spatial scale (**Figure 5A**). The highly suitable habitats (with a suitability index of 0.590–1) were mainly found in the North China Plain, Huang-Huai-Hai Plain, and parts of Liaoning and Jilin provinces in China, as well as Korea and parts of Japan. For *G. max*, the range of suitable habitats (**Figure 5B**) (with a suitability index of 0.296–1) was similar to that of *A. glycines*. However, the highly suitable habitats (with a suitability index of 0.648–1) were predominantly located in Korea, most of Japan, and scattered parts of China. Consequently, the overlaying analysis showed that the ranges where the two species’ habitats overlap primarily extended from South China to Northeast China in East China, Korea, and most of Japan (**Figure 5C**).

### Potential suitable habitats of four enemy species

**Figure 6:**
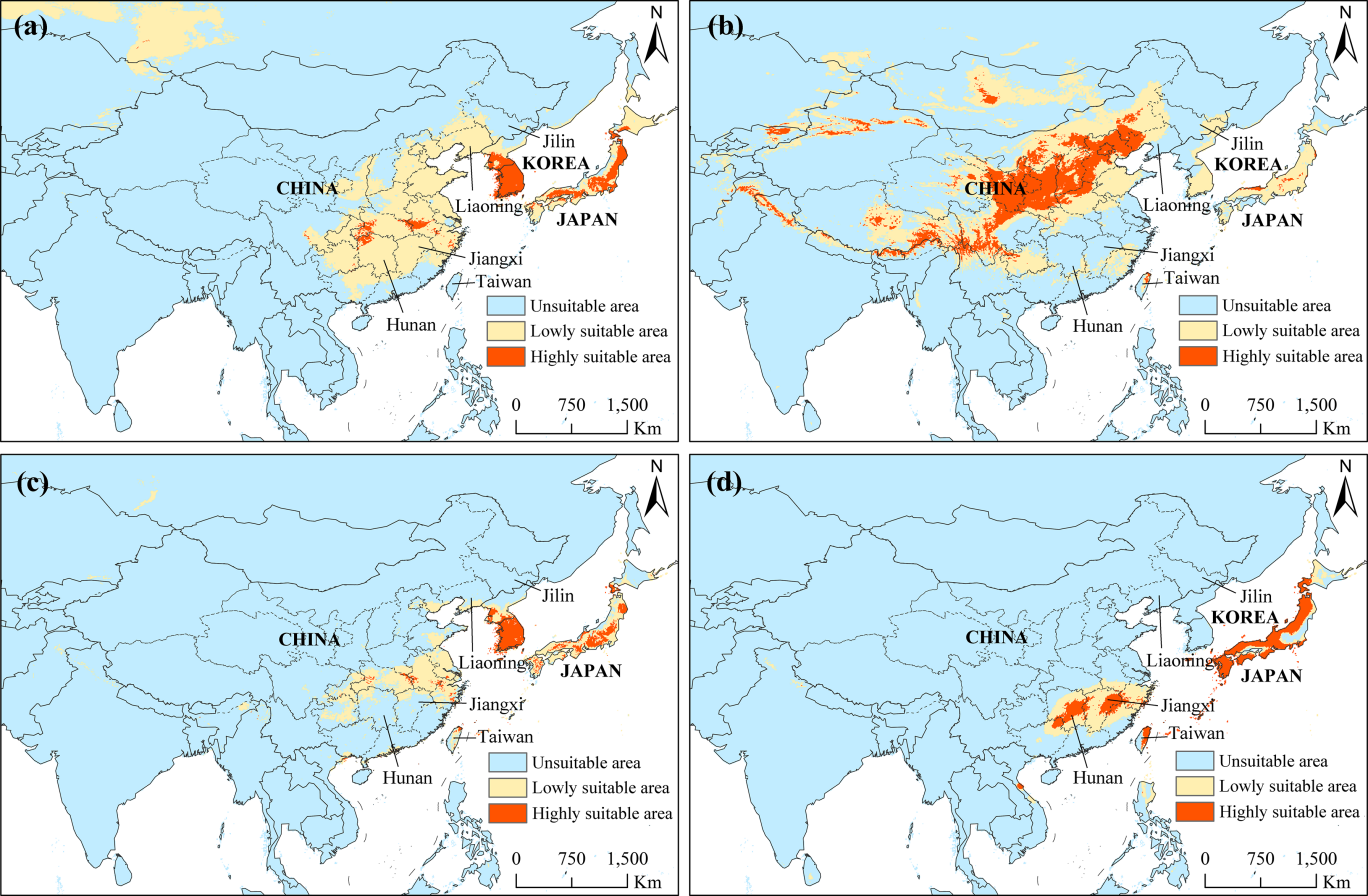
Potential suitable habitats of four natural enemy species. The Countries (in bold) and five provinces from China mentioned in the text are marked. **(a)** *Harmonia axyridis*. **(b)** *Chrysopa pallens*. **(c)** *Episyrphus balteatus*. **(d)** *Lysiphlebia japonica*.

The suitable habitats of four enemy species of *A. glycines* exhibited different distribution patterns (**Figure 6**). The suitable habitats of *H. axyridis* (with a suitability index of 0.133–1) were primarily distributed across East China, Korea, and Japan (**Figure 6A**). The highly suitable habitats (with a suitability index of 0.567–1) were mainly found in Korea, most of Japan, and a little parts of central China. For *C. pallens*, the suitable habitats (with a suitability index of 0.401–1) were mainly in most of China, including North China and parts of northwest and southwest China, as well as most of Korea and Japan. The highly suitable habitats (with a suitability index of 0.701–1) were mainly found in North China, and scattered parts of northwest and southwest China. The suitable habitats of *E. balteatus* (with a suitability index of 0.231–1) were significantly contracted compared to the two species described above, primarily distributed in Korea, most of Japan, and parts of central China. The highly suitable habitats (with a suitability index of 0.616–1) were mainly found in Korea, and parts of Japan. For *L. japonica*, the suitable habitats (with a suitability index of 0.343–1) were mainly in South China, and Japan. The highly suitable habitats (with a suitability index of 0.672–1) were mainly found in the provinces of Hunan, Jiangxi and Taiwan in South China, and most of Japan.

### The overlaying ranges of *A. glycines* and its enemies

**Figure 7:**
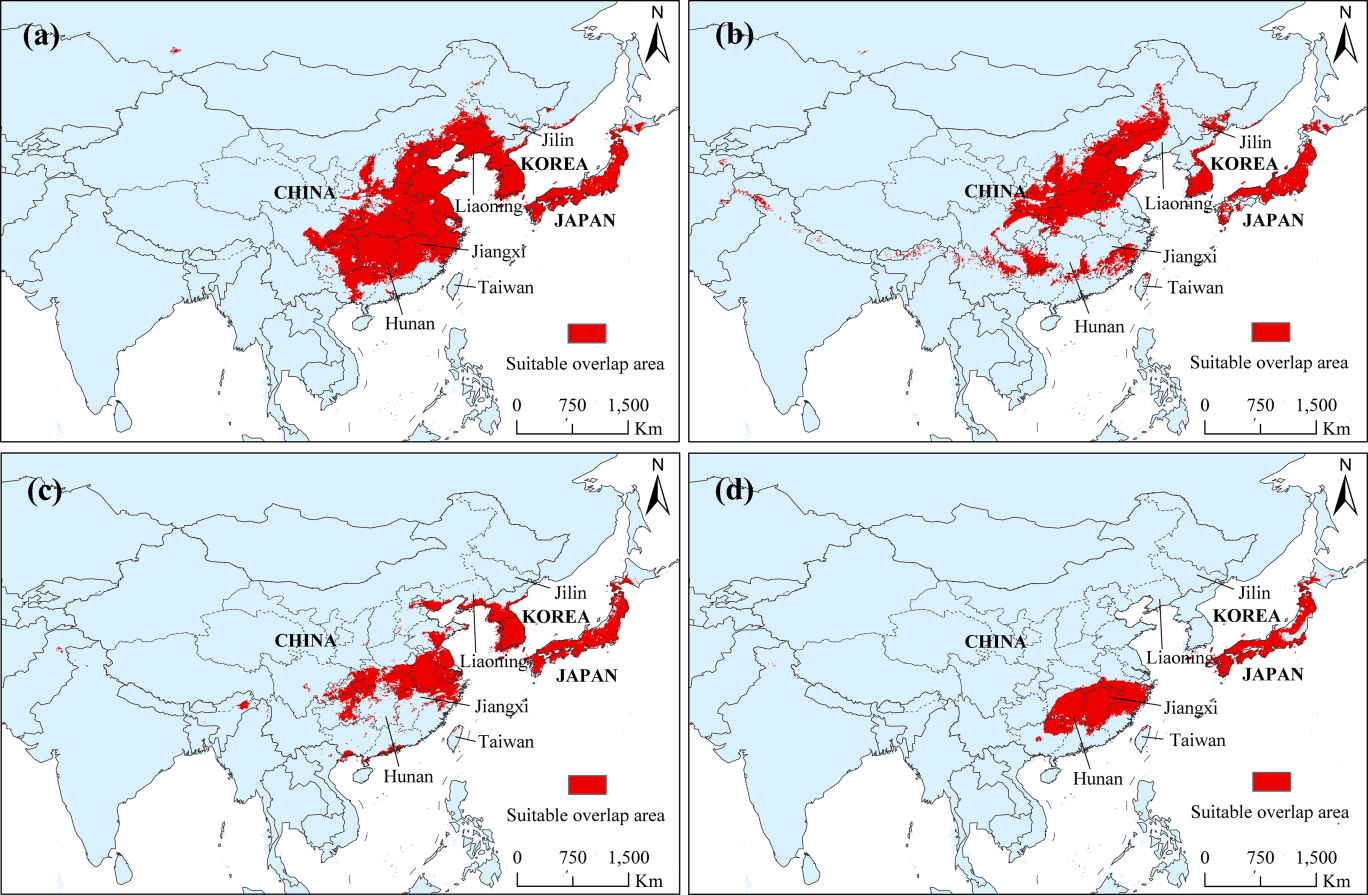
The overlaying ranges of *A. glycines* and its selected natural enemies. The Countries (in bold) and five provinces from China mentioned in the text are marked. **(a)** *Aphis glycines* + *Glycine max* + *Harmonia axyridis*. **(b)** *Aphis glycines* + *Glycine max* + *Chrysopa pallens*. **(c)** *Aphis glycines* + *Glycine max* + *Episyrphus balteatus*. **(d)** *Aphis glycines* + *Glycine max* + *Lysiphlebia japonica*.

To delineate the overlaying suitable ranges between *A. glycines* and its natural enemies, the predicted suitable habitats for *A. glycines* in the presence of *G. max* was superimposed onto the potential suitable habitat of each of the four natural enemies (**Figure 7**). The range of overlap with *H. axyridis* (**Figure 7a**) was widely distributed across East China, Korea, and Japan. In contrast, the overlaying range with *C. pallens* (**Figure 7b**) was primarily in most of North China, parts of South China, Korea, and Japan. With *E. balteatus*, the range of overlap (**Figure 7c**) was mainly distributed in central China, Korea, and Japan. The overlaying range with *L. japonica* (**Figure 7d**) was mainly concentrated in parts of South China, and most of Japan.

## Discussion

In this study, through various overlaying analyses, we identified the overlaying ranges of suitable habitats of *A. glycines* and its four representative natural enemies. The results could effectively facilitate the selection and application of biological control agents (natural enemies) for the regional management of this economically important pest within its native range.

The distribution patterns of suitable habitats for *A. glycines* in China, Korea, and Japan, as predicted by our study, is generally consistent with previous records (Fang et al., 2018; Wu et al., 2004). Besides, the predicted distribution patterns of suitable habitats for *A. glycines* are generally in agreement with those predicted by Ma et al (Ma et al., 2022). based on the MaxEnt modeling method. However, there are subtle differences in the area of highly suitable habitats, where the results of Ma et al (Ma et al., 2022). show broader coverage across East China, Korea, and Japan. This difference can be attributed to the different model parameters used. For instance, in our study, the parameters, FCs and RM, and their combinations are optimized given that their combinations can significantly influence model performance and transferability (Muscarella et al., 2014; Zhao et al., 2024). Additionally, we used the MTSPS logistic threshold value as a threshold to define suitable and unsuitable areas, in contrast to the 10 percentile training presence used in Ma et al (Ma et al., 2022).

Temperature and precipitation are recognized as crucial factors influencing the distribution of species, likely due to their strong correlation with energy and water availability (Barbet-Massin et al., 2012; Bell et al., 2014; Rossi and Rasplus, 2023; Trisos et al., 2020; Zhang et al., 2022). Among the bioclimatic factors examined, BIO04 (temperature seasonality) and BIO02 (mean diurnal range) exhibit relatively higher contributions to the modeling, accounting for up to 65% collectively. This suggests that temperature has a greater impact than precipitation on the habitat distribution of *A. glycines*. Both BIO04 and BIO02 reflect the values of rhythmic changes in temperature. Response curves indicate that *A. glycines* prefers habitats with a relatively distinct temperature difference within a day or across different seasons to some extent. For instance, when BIO04 is approximately 1200, and BIO02 is around 11, *A. glycines* has the highest presence probability. This outcome aligns with the prediction map, which indicates that the highly suitable habitats are primarily located between 30°N and 45°N, as also indicated by Ma et al (Ma et al., 2022), where show higher temperature difference than regions with lower latitudes.

The Asian Harlequin ladybird, *H. axyridis*, is a generalist predator commonly found in agricultural ecosystems. It is known for preying on aphids (Di Cola et al., 2017; Sui et al., 2025). It has been frequently studied and utilized for managing *A. glycines* in soybean fields (Koch and Costamagna, 2017; Liu et al., 2012; Wu et al., 2004; Xue et al., 2009). Native to East Asia, this insect is considered an invasive alien species in America and Europe for biological pest control purposes (Bidinger et al., 2012; Ongagna et al., 1993). In East Asia, *H. axyridis* is predominantly found in East China, Japan, and Korea (Bidinger et al., 2012), which aligns with our predictions. However, our results, obtained using the optimized MaxEnt model, indicate a slightly smaller range of suitable habitats compared to the findings of Bidinger et al (Bidinger et al., 2012). who used a non-optimized MaxEnt model. Specifically, part of North China is not predicted to be a suitable habitat in our study. Moreover, the distribution pattern of highly suitable habitats largely corresponds with that of Bidinger et al (Bidinger et al., 2012). primarily including Japan and Korea. In China, our predictions suggest that the highly suitable habitats are limited and sparsely distributed in central China, whereas Bidinger et al (Bidinger et al., 2012). predicted them to be sparsely distributed in South China. The potential distribution pattern revealed herein indicates that, in East Asia, *H. axyridis* can serve as a natural enemy over a wide range, especially in Japan and Korea. By overlaying and analyzing the suitable habitats of the pest and *H. axyridis*, the overlaying range in central and East China, Japan, and Korea suggest that *H. axyridis* can serve as a biological control agent for *A. glycines* management, especially in Korea and most of Japan, which are highly suitable for both species.

The green lacewing *C. pallens* is a commonly found and widely used predator of various pests, including *A. glycines* (Dai and Zu, 1997; Liu and Zhao, 2007; Radev, 2022; Wang et al., 2023; Xu et al., 2019). Unlike other green lacewings, both its larvae and adults prey on insect pests (Du et al., 2025; Liu et al., 2013; Zhang et al., 2004). Moreover, its high control potential is supported by a broad diet, a high predation rate, strong adaptability, and high egg production (approximately 2000 eggs per female) (Wang et al., 2022). To date, it has been reported to have a wide distribution range across Asia and Europe, and in Asia, it is the dominant species, widely distributed in China and also found in Japan and Korea (Wang et al., 2022; Yang et al., 2018). This distribution is generally consistent with our projections, with suitable habitats covering most of China, and most of Korea and Japan, in the study area. The region where *C. pallens* is suitable suggests its potential for biological control in the development of sustainable and integrated pest management strategies. For *A. glycines* management, the overlaying analyses indicate that the joint suitable habitats are majorly distributed in most of North China, parts of South China, Korea, and Japan, suggesting these areas may be more suitable for releasing *C. pallens* to control *A. glycines*.

The Marmalade Hoverfly, *E. balteatus*, is one of the most abundant beneficial insects. As a generalist predator, it offers protection against agricultural pests, including aphids (Dong et al., 2004; Hawkes et al., 2023; Jiang et al., 2025a; Jiang et al., 2025b). In soybean fields, *E. balteatus* has been studied as an important natural enemy of *A. glycines* (Dai and Zu, 1997; Wu et al., 2004). It has been reported that this insect has a distribution spanning from Asia and Europe to Africa and Australia, with a particularly dominant presence in China (Jiang et al., 2025a; Sadeghi, 2008; Yang et al., 2002). In this study, for the first time, we predicted that the suitable habitats are primarily in Korea, most of Japan, and parts of central China within the study area. This suggests its suitability for application in pest control. Through overlaying analysis, the overlaying suitable ranges of *E. balteatus* and *A. glycines* are distributed in central China, Korea, and Japan, indicating that these areas can utilize *E. balteatus* as a potential biological control agent for *A. glycines* management. In particular, Korea and parts of Japan are highly suitable for both species.

The *L. japonica* is an endophagous parasitoid, and the association between this insect and its host *Aphis gossypii* have been intensively investigated (Gao et al., 2021, 2020; Li et al., 2024; Zhou et al., 2025). As a closely-related species of *A. gossypii*, *A. glycines* has also been parasitized by *L. japonica* (Gao, 1994; Liu and Zhao, 2007). However, the delineation of areas suitable for *L. japonica* release to control *A. glycines* has not been investigated at a spatial scale. The overlaying analysis of the suitable habitats of *A. glycines* and *L. japonica* shows that their overlaying ranges are primarily distributed in parts of South China and most of Japan, indicating that these areas have a higher possibility of effectively controlling *A. glycines*.

Based on the overlaying results and climate environment, we propose targeted biological control strategies for *A. glycines*. *Harmonia axyridis* is recommended as the preferred natural enemy for large-scale release in East China, Korea, and Japan due to its wide niche overlap with *A. glycines* and strong environmental adaptability. *Chrysopa pallens* is suitable for release in areas with moderate annual mean temperatures (e.g., central and southern East China) to maximize its predation efficiency. *Episyrphus balteatus* should be released in areas with sufficient precipitation in the warmest quarter, which are consistent with the suitable areas of *G. max*, to ensure its food supply and activity. For *L. japonica*, it is recommended to release it in areas with adequate precipitation in the driest quarter, such as parts of South China and most of Japan. These practical suggestions provide a scientific basis for the regionalized and targeted biological control of *A. glycines*.

Despite the efforts to enhance the rigor and practicality of this study, several limitations should be acknowledged, which provide clear directions for future research and help contextualize the interpretation of the study’s results. First, the transferability of SDM results to other non-study regions those with climate similarity, and field validation should be conducted to evaluate the actual pest management outcomes. Second, sampling bias, particular for *L. japonica*, may led to optimistic overestimation of AUC and TSS values. To strengthen the validity of model performance assessments beyond AUC and TSS, the adoption of additional spatially independent evaluation methods (e.g., environmental clustering) would enhance the robustness of the results. Third, the overlaying analysis is presented largely as qualitative map interpretation, and to quantify overlaying area (km²) may be more informative to guide biological control. Fourth, it should be acknowledged that niche overlap only reflects the spatial consistency of suitable habitats between *A. glycines* and its natural enemies, and does not account for the temporal synchronization of their occurrence or variations in predation/parasitism efficiency (e.g., those induced by climate change). Moreover, this study relied solely on bioclimatic variables to model habitat suitability, ignoring non-climatic factors (such as land use type, agricultural management practices, and planting density) that also directly influence the distribution of *A. glycines*, its host *G. max*, and their natural enemies (Macfadyen et al., 2018). Therefore, in future research, future climate change scenarios and non-climatic variables should be incorporated to SDMs to improve the accuracy and ecological rationality of habitat suitability predictions, thereby facilitating the targeted biological management of *A. glycines*.

## Conclusions

This study aims to provide science-based guidance for the regionalized biological control of *A. glycines*, a destructive soybean pest, to support sustainable agricultural development. Based on the optimized MaxEnt model findings, we put forward targeted biological control strategies for different natural enemies. These suggestions are highly relevant to agricultural planning, as they enable local authorities to implement targeted, cost-effective biological control instead of widespread chemical pesticide use. For public policies, we suggest integrating these habitat suitability and natural enemy release strategies into regional pest management frameworks, promoting eco-friendly agriculture. Future research should incorporate non-climatic factors and climate change scenarios, conduct field validation, and improve model transferability to enhance the practicality of these strategies, which ultimately contribute to reducing pest-related economic losses and advancing sustainable soybean production.

## Data Availability

The original contributions presented in the study are included in the article/**Supplementary Material**. Further inquiries can be directed to the corresponding authors.
