# Supplementary figures and images for: Habitat suitability for the soybean aphid, Aphis glycines, and its natural enemies: implications for biological control and soybean protection
Source: Front Plant Sci. 2026 Jun 3;17:1845163. doi: 10.3389/fpls.2026.1845163 (PMC13272387; doi:10.3389/fpls.2026.1845163)

## Slide 1
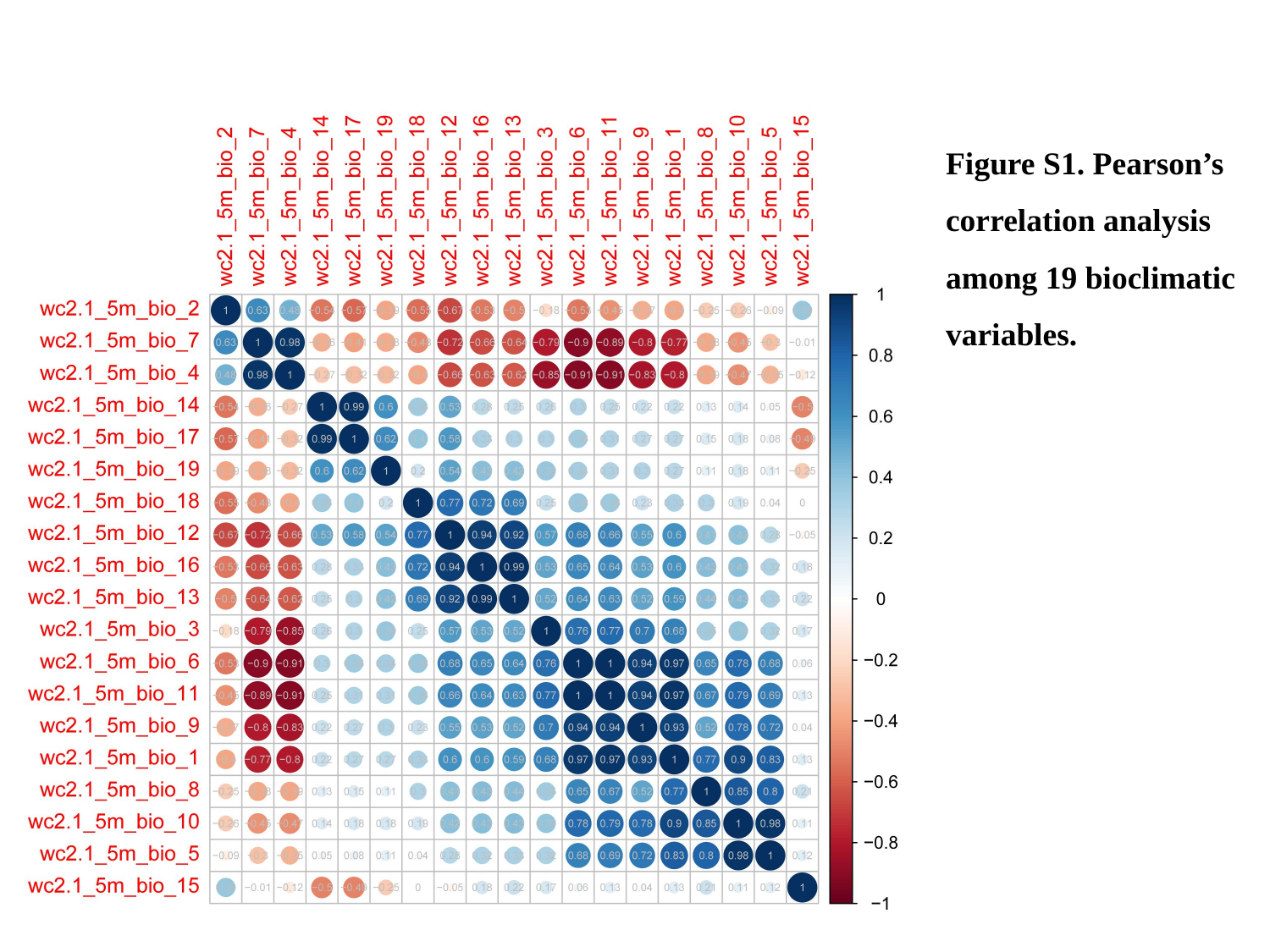

Figure S1. Pearson’s correlation analysis among 19 bioclimatic variables.

Supplement: Supplementary file 1 [file Presentation1.pptx]
