# Supplementary material for: Habitat suitability for the soybean aphid, Aphis glycines, and its natural enemies: implications for biological control and soybean protection
Source: Front Plant Sci. 2026 Jun 3;17:1845163. doi: 10.3389/fpls.2026.1845163 (PMC13272387; doi:10.3389/fpls.2026.1845163)
Supplement: Supplementary file 3 [file Table2.docx]

**Table S2. The 19 bioclimatic variables considered in this study**

| **Bioclimatic variable** | **Description** |
| --- | --- |
| BIO01 | Annual mean temperature (°C) |
| BIO02 | Mean diurnal range (mean of monthly (max temp-min temp)) (°C) |
| BIO03 | Isothermality (bio2/bio7) (× 100) |
| BIO04 | Temperature seasonality (standard deviation ×100) |
| BIO05 | Max temperature of warmest month (°C) |
| BIO06 | Min temperature of coldest month (°C) |
| BIO07 | Annual temperature range (bio5–bio6) (°C) |
| BIO08 | Mean temperature of wettest quarter (°C) |
| BIO09 | Mean temperature of driest quarter (°C) |
| BIO10 | Mean temperature of warmest quarter (°C) |
| BIO11 | Mean temperature of coldest quarter (°C) |
| BIO12 | Annual precipitation (mm) |
| BIO13 | Precipitation of wettest month (mm) |
| BIO14 | Precipitation of driest month (mm) |
| BIO15 | Precipitation seasonality (coefficient of variation) |
| BIO16 | Precipitation of wettest quarter (mm) |
| BIO17 | Precipitation of driest quarter (mm) |
| BIO18 | Precipitation of warmest quarter (mm) |
| BIO19 | Precipitation of coldest quarter (mm) |
